# The Way to Industrial Health

**Published:** 1920-11-13

**Authors:** 


THE PUBLIC WELL-BEING—(*continued*).

### The Way to Industrial Health.

The direct bearing of working and living conditions on health is a platitudinous subject, but a long road has yet to be travelled before the realisation of it is widely translated into practice. Things are certainly moving, as we have had occasion to show, not only on the purely health side, but also on the amenities side, which has a close relation to health. An interesting description is given in the commercial supplement of the *Manchester Guardian* of a welfare scheme as it is worked in a firm engaged in the production of jams and other preserves. This firm organised a welfare department, to eliminate, as far as possible, the leaving of employees on account of dissatisfaction with working conditions.

The scheme adopted was based on a broad conception of the meaning of "efficiency." Every employee on entering the firm's service is presented with a booklet containing the rules of the factory. In addition to details of hours and regulations, there are appeals for loyalty and co-ordination on the part of the employees. A forceful note is struck in one headed "Co-operate or Quit!" The directness of this appeal is softened somewhat by the subsequent account of the efficiency pay which is awarded monthly, the suggestion rewards, and the marriage gifts, ranging from £1 to £5, according to length of service.

Bathrooms, in the proportion of one to every fifty employees, are provided on the factory premises, and are open for use during working hours. One half-day a week and two half-hours daily are spent in cleaning machinery and utensils. A works committee, with departmental sub-committees, exists to make recommendations for the smoother running of the factory and the elimination of points of danger. There are holidays savings schemes and a sick benefit club, towards which the firm makes a grant from profits, besides paying interest on deposits in the former.

The canteen is large and well furnished. Dinners cost from 4d. to 7d., according to amount of wage earned, and teas, for overtime workers, 2d. or 3d. The dining hall is used also for social activities. The factory has its band and a choir, a dramatic society, and a pierrette troupe. Dances and "socials" are of frequent occurrence. A factory library has been formed, and evening classes are held.

All this results, it is found, in an extremely low labour "turnover."

